# Application of IgY antibodies in passive immunization of aquaculture animals

**DOI:** 10.3389/fphys.2025.1701782

**Published:** 2025-11-20

**Authors:** Hui-long Qiu, Xiao-min Jin, Xiao-mei Zhang, Feng-qin Wang, Jia-qiang Huang, Lian-shun Wang

**Affiliations:** 1 College of Marine Resources and Environment, Hebei Normal University of Science and Technology, Hebei Key Laboratory of Ocean Dynamics Resources and Environments, Qinhuangdao, China; 2 Zaozhuang Animal Husbandry and Fishery Development Center, Zaozhuang, China; 3 Key Laboratory of Precision Nutrition and Food Quality, Ministry of Education, Department of Nutrition and Health, China Agricultural University, Beijing, China; 4 School of Fisheries and Life Science, Dalian Ocean University, Dalian, China

**Keywords:** IgY antibodies, passive immunization, Aquaculture animals, IgY, Aquaculture

## Abstract

Chicken Egg Yolk Immunoglobulin (IgY) is a specific antibody found in egg yolk, offering several advantages, including low production cost, pollution-free processing, and no drug resistance. IgY as a passive immunotherapy agent in the field of aquaculture, the focus is on the prevention and control of common aquatic diseases, including vibriosis (*Vibrio parahaemolyticus, Vibrio harveyi, Vibrio splendidus*), bacterial septicemia (*Aeromonas hydrophila, Aeromonas salmonicida*), and viral diseases (*Nervous Necrosis Virus, White Spot Syndrome Virus*). The administration of specific IgY via feed, oral intake, immersion, or injection has been shown to significantly enhance antibody levels and phagocyte activity in shrimp, fish, sea cucumbers, and other aquatic animals. This approach has been demonstrated to reduce pathogen colonization, enhance immune performance, and decrease infection incidence and mortality rates. In the context of sustainable aquaculture development, IgY has emerged as a significant biological immunomodulator, with the potential to replace traditional antibiotics and control diseases in aquatic animals. This review summarizes the fundamental structure differences from IgG, physicochemical properties, and preparation methods of IgY, with a focus on its passive immunotherapy application progress in disease prevention, treatment, and immunization for freshwater and marine aquaculture animals. Finally, the positive effects of IgY on the quality and safety of aquatic products were discussed, with the aim of conducting in depth research on IgY and its widespread application in aquaculture.

## 1 Introduction

Aquaculture products are a vital source of food and nutrition for the global population, as well as providing economic value. In recent years, the world’s *per capita* supply of aquatic products has steadily increased, largely thanks to the rapid growth of the aquaculture industry. Currently, aquaculture accounts for around 50% of the total amount of aquatic products consumed by humans, with both marine and freshwater aquaculture playing a crucial role in ensuring adequate nutrition and food security for everyone (Norman et al., 2019). China is widely recognized as a major global fisheries power, being one of the few countries where aquaculture production exceeds that of capture fisheries. For 20 consecutive years, China has ranked first worldwide in total fisheries production and aquaculture output (Mondal and Lee, 2025). However, as aquaculture intensifies and expands, high-density farming and environmental pollution are leading to frequent disease outbreaks and increased morbidity among aquatic animals, causing substantial economic losses to the industry. Conservative estimates suggest that farmed shrimp experience annual production losses of around 40% (>$3bn) due to bacterial pathogens, and traditional preventive measures are ineffective (Stentiford et al., 2012). Pathogenic bacteria can damage fish in multidimensional and systemic ways. For example, *Saprolegnia* can invade fish epidermal cells, causing a disease called saprolegniasis, while *Aeromonas hydrophila* can penetrate fish gills, inducing gill rot as well as bacterial enteritis and meningitis. The harm caused to fish farming is characterized by its explosive and contagious nature. Antibiotics have historically been widely used in aquaculture to prevent and treat bacterial diseases. However, around 90% of antibiotics used in aquaculture are administered as growth promoters and prophylactics, rather than for specific treatments against pathogenic microbial infections, and prolonged antibiotic use has led to bacterial resistance and drug residue issues in aquatic products, posing serious potential risks to human health (Adenaya et al., 2023). Data indicate that sulfadiazine, sulfamethoxazole, trimethoprim, erythromycin-H_2_O, and amoxicillin were the most frequently detected antibiotics, with sulfadiazine reaching a peak concentration of 25,000 ng/L, resulting in pathogen resistance rates exceeding 60% for pathogens such as *Vibrio* and *A. hydrophila* (Salma et al., 2025). Overall, the sustainable development of the aquaculture industry is constrained by multiple factors. Developing strategic aquaculture plans, improving disease prevention and control measures for aquatic animals and expanding the green, healthy production of aquaculture products are crucial for global food security and more sustainable practices (Peeler and Ernst, 2019; Ma et al., 2025). Therefore, there is an urgent need to develop green, efficient, affordable and readily accessible disease-specific therapeutic products for aquatic animals.

Chicken Egg Yolk Immunoglobulin (IgY) are antibodies found in the blood of birds, amphibians and reptiles. It has functions including resistance to bacterial and viral infections, binding to antigens, and neutralizing toxins. Compared to traditional antibodies, IgY exhibits greater specificity and safety, and a higher yield. Furthermore, it does not contaminate aquaculture water bodies. Unlike antibiotics and chemical disinfectants, IgY leaves no drug residues and does not induce drug resistance. Therefore, developing IgY vaccines for aquaculture is highly valuable. This review introduces the biological structure and physicochemical properties of IgY, summarize the principles of IgY vaccine preparation and outlines its application in freshwater and marine aquaculture. Finally, it considers the potential and obstacles to promoting IgY in the aquaculture sector. The aim of this review is to provide a reference for future IgY research and its broader application in aquaculture.

## 2 Biological structure and physicochemical properties

### 2.1 Structure and function of IgY

**FIGURE 1:**
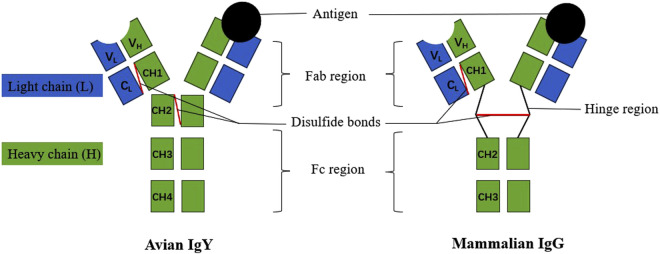
Structure of IgY and IgG.

IgY is a typical four chain structural model formed from two identical heavy (H) chains and two identical light (L) chains. These chains are interconnected through non-covalent bonds (hydrophobic interactions and hydrogen bonds) and covalent bonds (disulfide bonds), creating a symmetrical, three-dimensional Y-shaped structure. Each H chain consists of variable (VH) and constant (CH1, CH2, CH3 and CH4) regions, while each L chain consists of variable (VL) and constant (CL) regions (Zhang et al., 2017). IgY and IgG have some structural and functional similarities, and the former is traditionally considered to be an evolutionary precursor of the latter, but there are some differences between the two (Figure 1). IgY has a longer heavy chain and a higher molecular weight of 180 kDa compared to 150 kDa for IgG. IgY also has more glycosylated side chains and constant structural domains which are more hydrophobic and inhibit the hydrolysis of proteins catalysed by proteinases (Lee et al., 2021). In addition, the IgY molecule lacks a hinge region between CH1 and CH2. This region is unable to undergo conformational changes and is less flexible. IgY also differs from other immunoglobulins in terms of amino acid sequences and glycosylation patterns in some regions, and has additional CH4 structural domains.

The VH and VL regions of the variable zone are responsible for binding specifically to antigens, and their high diversity enables them to accommodate a wide range of different antigens. In contrast, the constant regions (CH, CL) are more conserved among different types of antibodies and are responsible for stability and function. For instance, the CH3 and CH4 regions facilitate the homotypic polymerization of antibodies, forming multimers. The CH region of IgY contains glycosylation sites, and glycosylation plays an important role in the antibody’s stability and solubility. The N-glycosylation chain of IgY contains two glycostructures, namely, the high mannose chain and the composite glycan chain, and is predominantly a high mannose chain, with the presence of sialic acid and galactose modifications at the end of the glycan chain (Sheng et al., 2017). In addition, IgY has two functional regions, Fab and Fc, but no hinge region, with a short region rich in proline and glycine residues between the Fab and Fc segments. The Fab region (VH, CH1, VL and CL) is responsible for antigen binding, whereas the Fc region (CH2, CH3 and CH4) is involved in immune effects such as antibody dependent cell mediated cytotoxicity (Calvert et al., 2024). The structure of IgY determines their functions, including antigen-binding specificity and immune effector functions.

Additionally, compare to mammal antibody, the application of IgY contains a number of advantages. IgY is similar to other antibodies in that its variable region can bind specifically to antigen, while its Fc region has an immunomodulatory function. The Fc binds specifically to three characterized Fc receptors, such as chicken Ig-like receptor AB1 (CHIR-AB1), the chicken yolk sac IgY receptor (FcRY) and Gallus gallus Fc receptor (ggFcR) (Zhang et al., 2017; Okamoto et al., 2024). Due to the different residue groups and structural conformation of IgY, its Fc region is unable to interact with mammalian Fc receptors (IgG-FcγR, IgE-FcεR), rheumatoid factor (RF), complement factor (CF), *Staphylococcal* protein A, *Streptococcal* protein G, and *Peptostreptococcal* protein L (Lee et al., 2017) (Table 1). As IgY cannot bind to the aforementioned mammalian immunomodulatory proteins, it does not interact with mammalian immune complexes during immunoassays or therapy. This reduces the effects of endogenous interference and cross-reactivity. This offers many advantages and conveniences when using IgY in aquaculture.

**TABLE 1:** Interbinding regions of IgY and IgG with specific receptors or ligands, adapted from Lee et al. (2017).

Receptors or ligands	IgY	IgG
CHIR-AB1	CH3-CH4 interdomain	No
ggFcR	CH2-CH3 interdomain	No
FcRY	CH3-CH4 interdomain	No
IgG-FcγR	No	CH2-CH3 interdomain, CH2 hinge region
IgE-FcεR	No	CH2-CH3 interdomain, CH2 hinge region
Rheumatoid factor (RF)	No	CH2-CH3 interdomain
Complement factor (CF)	No	CH2-CH3 interdomain, CH2 hinge region
Staphylococcal protein A	No	CH2-CH3 interdomain, CH2 hinge region
Streptococcal protein G	No	CH2-CH3 interdomain

### 2.2 Physical and chemical properties of IgY

IgY exhibits strong thermal stability and resistance to acid and degradation by specific enzymes. The optimum temperature and time were found to be 30 °C, up to 8 h for IgY antibodies. IgY is almost twenty-fold more stable than IgG at 60 °C for up to 8 h (Gandhi and Alshehri, 2021). This is attributed to the important role of the CH4 domain in the heavy chain of IgY in molecular stability. IgY was quite stable at pH 5-7. Irreversible inactivation of IgY was observed at pH below 4, and proceeded rapidly at pH below 3. In a 50% aqueous sorbitol solution, an acid-induced inactivation was almost completely suppressed at pH 3 (Lee K. A. et al., 2002). Studies have shown that the immune activity of IgY in egg yolk remains relatively stable within the temperature range of 20 °C–50 °C. However, at 37 °C and a pH of 2–4 or 8–10, the activity of IgY changes significantly. Pepsin can hydrolyze IgY when the pH is 2.0 or 3.0, but the hydrolytic effect of pepsin on IgY is significantly reduced when the pH is between 4 and 6 (Xu et al., 2009). N-glycosylation protect the proteins against proteolytic degradation, aggregation, and thermal denaturation through maintaining optimal conformations (Zhou and Qiu, 2019). IgY possesses excellent immunological properties, it is non-toxic and has no side effects, drug resistance or drug residue issues. As such, it is an excellent, green, efficient and environmentally friendly alternative to antibiotics or chemical drugs, holding significant value for the healthy and sustainable development of the aquaculture industry.

## 3 Preparation and mechanism of action of IgY for aquaculture

### 3.1 IgY preparation

**FIGURE 2:**
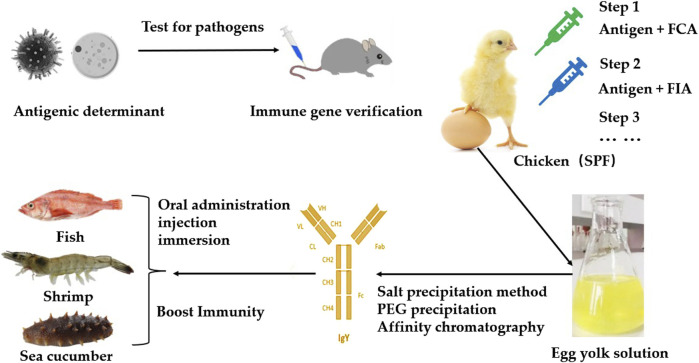
Principles for Preparing Aquatic IgY. SPF, Specific Pathogen Free; FCA, Focis complete adjuvant; FIA, Freund’s incomplete adjuvant; PEG, Polyethylene glycol.

IgY is primarily found in egg yolk, accounting for around 10% of the total protein content of the yolk. IgY, which can be isolated from egg yolk, can be administered orally or by injection, or used in immersion treatments, to prevent and treat various bacterial and viral diseases in aquatic animals (Redwan et al., 2021). When laying hens are immunized with specific antigens, B lymphocytes in the humoral immune system are activated and secrete IgY antibodies. These antibodies are then transported through the hen’s ovaries and oviduct into the egg yolk. With repeated immunization boosters, the titer of the specific IgY in the yolk peaks 3–4 weeks after immunization and can be sustained for 6–8 months. To preserve IgY activity, lipids and impurity proteins can be removed using ammonium sulphate precipitation (Pringels et al., 2018), polyethylene glycol precipitation (PEG) (Patel et al., 2025; Isah et al., 2025) or immunoaffinity chromatography (Diogo et al., 2024). Crude IgY requires further purification and testing to ensure compliance with aquaculture safety, efficacy and stability requirements (Figure 2). Finally, granules, powders, and liquids are prepared for oral administration, immersion, and injection, respectively, based on the aquatic species and disease type.

For enhancing the therapeutic and preventive functions of IgY antibody administration, a scientific and rational IgY coating and formulation (in the form of pellets, powder, or liquid) is crucial (Yakhkeshi et al., 2025). In fish, shrimp and sea cucumber farming, IgY has significant potential for diagnosing, preventing and treating bacterial and viral diseases, making it a valuable immunobiological tool for aquaculture applications (Capotă et al., 2025). Unlike antibiotics and chemical disinfectants, IgY does not pose a risk of chemical residue and does not induce pathogen resistance, thus aligning with the contemporary focus on green and healthy aquaculture practices.

### 3.2 IgY immune mechanism

IgY has good biological immune activity and is very suitable for the prevention and control of aquatic animal diseases. To date, IgY has been widely applied in aquaculture research targeting various pathogens, demonstrating its immune function through agglutination, adhesion blocking, toxin neutralization, and phagocytosis regulation (Zhang L,P. et al., 2024). In order to replicate and complete the infection process by producing toxins within the body, pathogenic bacteria that enter the host must adhere to the host’s intestinal mucosal cells. Research indicates that the primary mechanisms by which specific IgY suppresses pathogen activity are preventing adhesion and inhibiting growth. Key elements for bacterial colonization are specific components on the surface of Gram-negative bacteria, such as outer membrane proteins, lipopolysaccharides, fimbriae and flagella. IgY can recognize and bind to these components, thereby blocking bacterial adhesion to the host and ultimately inhibiting bacterial growth and expelling the bacteria from the body (Chalghoumi et al., 2009; Lee E. N. et al., 2002). The specific binding of IgY to bacteria may also alter cellular signaling cascades, thereby reducing toxin production and release. As an immunologically active component, IgY also has the potential to treat animal enteritis (Wang et al., 2024). Zhen et al. (2009) found that IgY improved the phagocytic activity of neutrophils against *Staphylococcus aureus*. In the presence of IgY, the phagocytic activity of macrophages or neutrophils against *Escherichia coli* increased significantly. These results suggest that IgY can enhance the phagocytic activity of immune cells. After pathogenic bacteria bind with IgY, the physical and chemical structure of the bacterial surface changes, rendering the bacteria more susceptible to phagocytosis.

In this study, IgY antibodies against live *shewanella xiamenensis* (LSX-IgY) and inactivated *shewanella. Xiamenensis* (ISX-IgY) were against major aquaculture Pathogens. The passive immunization protection rates of LSX-IgY and ISX-IgY against *Shewanella xiamenensis* were 63.64% and 72.73%, respectively, and the passive cross-protection rates against *A. hydrophila* were 50% and 71.43%, and the phagocytic activity of leukocytes was increased (Chen J. et al., 2025). Furthermore, the purified IgY was competent to neutralize and completely inhibited the *Red-spotted grouper nervous necrosis virus* (RGNNV) replication in the grouper fin cell line (GF-1), indicating that it was highly specific and effectively recognized RGNNV (Yi et al., 2018; Liu et al., 2021). Mandarin fish was fed diets supplemented with anti-MFNNV IgY, suggesting that oral administration of specific IgY could neutralize virus and reduce the immune responses as well as tissue pathological damage induced by the *Nervous necrosis virus* (NNV) (Liang et al., 2024). IgY inhibits the growth and reproduction of viruses by binding to the surface receptors of viral particles or by adhering to the viral capsid and affecting the fusion of the viral membrane to the cell surface.

In summary, IgY activates the immune response in fish by blocking mucosal pathways, activating humoral responses and enhancing cellular responses. Firstly, it blocks the invasion of pathogens at mucosal barriers (skin, gills and gut), thereby activating local mucosal immunity. Secondly, upon entering the humoral system, it activates the innate and complement immune systems, thereby inducing endogenous IgM production in fish. Finally, it enhances phagocytic function by activating cellular immunity while stimulating T-cell-mediated cytotoxic and helper functions.

## 4 Application of IgY in Aquaculture animals

### 4.1 Applications in freshwater aquaculture animals

The shift towards high-density farming has led to high mortality rates in freshwater aquaculture, primarily due to bacterial and viral diseases. This has caused substantial economic losses to the aquaculture industry (Irshath et al., 2023). For instance, diseases affecting shrimp, such as white spot syndrome and nervous virus disease, account for over 30% of losses in the global shrimp farming industry. It is imperative to develop sustainable and targeted disease prevention and control measures. IgY has functions that inhibit infection, enhance phagocyte activity and assist in disease prevention and control (Duman et al., 2025). Research indicates that administering inactivated *white spot syndrome virus* (WSSV) vaccines to laying hens via passive immunization through injection, oral administration or immersion with specific IgY antibodies effectively improves survival rates in *Procambarus clarkii* (Lu et al., 2009). A case study in anti-*Vibrio harveyi* IgY for *Fenneropenaeus indicus*, the parameters including coagulase activity, oxyhaemocyanin level, prophenoloxidase, intracellular superoxide anion production, lysozyme, phagocytosis and bacterial agglutinin had significantly increased (Kumaran et al., 2018; Kumaran et al., 2022). Additionally, specific IgY binds directly to *A. hydrophila*, causing agglutination and inhibiting bacterial growth. It enhances macrophage phagocytic activity, facilitating rapid bacterial clearance. Passive vaccination with anti-*A. hydrophila* IgY antibodies has been shown to have prophylactic or therapeutic effects against *A. hydrophila* in *Megalobrama amblycephala*, with survival rates reaching 60% (Qin et al., 2018). Numerous studies have shown that IgY improves the efficiency and economic viability of freshwater fish and shrimp farming (Table 2), playing a vital part in combatting pathogen invasion and enhancing survival rates in aquaculture.

**TABLE 2:** Applications of IgY in freshwater aquaculture animals.

Antibody types	Administration route/Dosage	Test animal/Weight	Pathogen	Efficacy of IgY	References
Anti-D4ORFs IgY	Oral administration 0.1%、0.3%、0.6%	*Carassius gibelio* 60 ± 10 g	*Cyprinid herpesviru*s-2 (CyHV-2)	Soaking is more effective than feeding Anti-D4ORFs IgY can effectively neutralize CyHV-2 *in vitro*	Sun et al. (2023)
Anti-WSSV IgY	Muscle injection 250 mg/mL	*Procambius clarkiaii* 10 g	*White spot syndrome virus* (WSSV)	With inactivated WSSV vaccine to neutralize WSSV, the challenged shrimp showed 73.3% survival	Lu et al. (2008)
Anti-*Vibrio* IgY	Oral administration 15%	*Litopenaeus vannamei* 4–5 cm length	*Vibrio harveyi、Vibrio parahaemolyticus*	The anti-Vibrio egg powders had an inhibiting effect on V. harveyi and V. parahaemolyticus *in vitro*. Lower mortality of infected zoeae, mysis, and postlarva was observed in groups fed with anti-Vibrio egg powders	Gao et al. (2016a)
Anti-OMPs IgY	Oral administration 10%	*Litopenaeus vannamei* 5.09 ± 1.16 g	The outer membrane proteins (OMPs) of *Vibrio parahaemolyticus*	The SOD in muscles of infected shrimp fed with specific IgY-included diets were significantly increased. Inhibited the growth of V. parahaemolyticus and introduced passive immunity to shrimp	Hu et al. (2019)
Anti-*E. cloacae* IgY	Oral administration 15%	*Macrobrachium rosenbergii* 0.19 ± 0.06 g	*Enterobacter cloacae*	Oral administration of specific IgY significantly improved the survival rate of E. cloacae-infected M. Rosenbergii, 67%	Gao et al. (2019)
Anti-*A. hydrophila* IgY	Immersion 0.5 g/L	*Carassius auratus Gibelio* 200–250 g	*Aeromonas hydrophila*	Haemoglobin concentrations, white and red blood cell numbers as well as the mortality of specific IgY-treated fish were significantly increased	Jin et al. (2013)
Anti-*A. salmonici*da IgY	Immersion 0.1 g/L	*Koi carp* 3.4–7 g	*Aeromonas salmonicida*	Prevent bacteria from invading the surface of fish and gills, and prevent contact with infectious ulcers	Gan et al. (2015)
IgY‐WSSV and IgY‐VP28	Oral administration 0.1% IgY‐VP28 plus 0.2% IgY‐WSSV	*Fenneropenaeus chinensis* (Osbeck, 1765) 25 g	*White spot syndrome virus* (WSSV)	IgY‐WSSV exerted a higher protection effect (71.5%) than IgY‐VP28 (63.7%). an increase in RPS (79.2%) was found on addition of 0.1% IgY‐VP28 plus 0.2% IgY‐WSSV,20 days	Fu et al. (2010)
Anti-CyHV-3 IgY	Oral administration 0.05%	*Carp* 10–25 g	*Cyprinid* herpesvirus 3 (CyHV-3)	Prevent common carp brain (CCB) cells from infection,the mortality of carp significantly decreased from 85% to 50%	Liu et al. (2014)
Anti-MFNNV IgY	Oral administration 33%	*Siniperca chuatsi* 16 ± 0.5 g	*Nervous necrosis virus* (NNV)	Neutralize virus and reduce the immune responses as well as tissue pathological damage induced by the NNV. Reduce the death rate by 36%, 7days	Liang et al. (2024)
Chicken *Enterococcus* faecalis- induced immunoglobulin Y	Oral administration 3% (0.50 g per fish per day)	*Oreochromis hybrid* 16.70 ± 3.07 g	Chicken *Enterococcus faecalis*	The pellet formulation of 50% egg yolk with an IgY concentration of 2.43 mg/g orally, with 3% body weight once a day, was the best in the experimental fish, 14days	Rizkiantino et al. (2023)
Anti-*V. vulnificus* IgY	Oral administration 10% intraperitoneal injection 15 mg/mL	*Danio rerio* 0.3–0.4 g	*Vibrio vulnificus*	Enhanced survival rates, with injected groups up to 90%, fed diets 71.43%, 7 days, reduce *Vibrio* and *Aeromonas* in the intestines	Chen et al. (2025b)
VF14355 IgY antibody	Intraperitoneal injection 30 μL	*Carassius auratus* 20 ± 1.0 g	*Vibrio fluvialis, Aeromonas hydrophila*	Serum immune factor levels, ACP, AKP, LZM were increased, the relative percent survival were 46.15% and 64.29%, 14 days	Xiao et al. (2025a)
VF17320 IgY antibody	Intraperitoneal injection 30 μL/30 μg	*Carassius auratus* 20 ± 1.0 g	*Vibrio fluvialis, Aeromonas hydrophila*	Increased activates the phagocytosis of chicken plasma leukocytes, the ACP, AKP, and LZM were significantly elevated, passive cross-immune protection rates were 73.33% and 60%, 14 days	Xiao et al. (2025b)
Anti-S. agalactiae IgY	Oral administration 10%	*Oreochromis niloticus* 50–70 g	*Streptococcus agalactiae*	Specific IgY decreased the proportion of *Streptococcus* and increased the diversity of the intestinal flora, reduced caspase activity and intestinal cell apoptosis	Zhang et al. (2024b)

### 4.2 Applications in marine animal aquaculture

Bacterial diseases such as vibriosis and viral diseases are predominant in marine aquaculture. The high salinity of seawater requires greater stability of IgY. Vibriosis is a common bacterial disease that affects marine farmed fish and echinoderms (Table 3), while viral nervous necrosis disease is one of the most prevalent and devastating viral infectious diseases affecting marine fish (Lu et al., 2025). Relevant data indicates that annual losses from aquaculture diseases exceed 50 billion, with over 20 billion of this coming from marine aquaculture. However, IgY can be prepared in large quantities and economically, and have potential value to against multiple bacteria in aquaculture (Liu X. et al., 2024). Immune protection of IgY against live and inactivated *Vibrio fluvialis* in fish (Xiao et al., 2025a). The IgY vaccines could protect the internal tissue structure integrity and reduce the apoptosis and DNA damage of kidney cells induced by bacterial infection (Xiao et al., 2025c). Studies indicate acute hepatopancreatic necrosis disease (AHPND), caused by a toxin-producing *Vibrio parahaemolyticus* strain, has become a serious threat to shrimp aquaculture. *Litopenaeus vannamei* fed with oral feed containing rPirA (anti-PirA-IgY) were protected from AHPND, with a survival rate as high as 87% (Nakamura et al., 2019). The outer membrane proteins (OMPs) PF1380 and ExbB, as well as the IgY antibodies of *Pseudomonas fluorescens*, have antioxidant and anti-inflammatory effects on fish (Liu et al., 2023). Due to environmental factors, the salinity, temperature and pH of seawater can fluctuate significantly, leading to instability in IgY activity and even denaturation. Therefore, improving the stability of IgY in marine aquaculture is crucial for precisely preventing and controlling diseases in marine aquaculture animals, and remains a key challenge for future research.

**TABLE 3:** Applications of IgY in marine aquaculture animals.

Antibody types	Administration route/Dosage	Test animal/Weight	Pathogen	Efficacy of IgY	References
Anti-*Vibrio* IgY	Oral prophylaxis 5%、10%	*Haliotis diversicolor supertexta* 10 mg	*Vibrio alginolyticus*	Fed with 5 or 10% anti-Vibrio IgY egg powders were in the range of 65%–70% 14 days post-V. alginolyticus challenge (1 × 10^6^ CFU)	Wu et al. (2011)
Anti-*V. splendidus* IgY	Intraperitoneal injection 10 mg/mL Immersion 1 g/L	*Apostichopus japonicus* 6.3 ± 0.24 g	*Vibrio splendidus*	Survival for intraperitoneal injection 80% and immersion 75%, 11 days enhanced the phagocytosis of coelomocytes for V. splendidus.	Li et al. (2016)
Anti-*S. marisflavi* AP629 IgY	Intraperitoneal injection 25 mg/mL oral prophylaxis 10%	*Apostichopus japonicus* 10.64 ± 0.84 g	*Shewanella marisflavi* AP629	Survival for intraperitoneal injection 77.5% and oral prophylaxis 57.5%. Enhance body cavity cell immunity and antioxidant performance	Xu et al. (2019)
Anti-*V. harveyi* IgY	Muscle injection 10 mg/mL Immersion 0.5 g/L	*Takifugu rubripes* 18.89 ± 1.13 g	*Vibrio harveyi*	Survival for muscle injection 80% and immersion 60%, 7 days. IgY enhanced the phagocytosis of *Vibrio* harveyi by macrophages	Zhang et al. (2021)
Anti-*E. tarda* 2CDM001 IgY	Oral prophylaxis IgY microencapsulated 5%	*Scophthalmus maximus* 56 ± 2 g	*Edwardsiella tarda* 2CDM001	Survival for 63.3%, 10 days. Significantly reduced IL-1β, IL-8, TNF-α and C3 transcript levels	Xu et al. (2020)
Anti-*V. anguillarum* IgY-encapsulated	Oral prophylaxis 15%	*Cynoglossus semilaevi* 15–20 g	*Vibrio anguillarum*	Survival rate is 70%, reduce bacterial invasion of the blood, liver, and kidneys	Gao et al. (2016b)
Anti-*V. parahaemolyticus* IgY	Muscle injection 14 mg/mL	*Rachycentron canadum* 380 g	*Photobacterium damselae subsp. piscicida*	Reducted the bacterial load in liver and spleen of *Rachycentron canadum*	Eto et al. (2019)
Anti-SGIV IgY	Oral prophylaxis 2%	*Epinephelus coioides* 5 ± 0.5 g	*Singapore grouper iridovirus* (SGIV)	The relative protection rate 40%, reducing the infection of target organ cells and attenuating the inflammatory response	Liu et al. (2024b)

## 5 The benefits of IgY on the quality and safety of aquatic products

In aquaculture, IgY is a green biopreparation that not only improves the health of aquatic animals and increases farming efficiency, but also directly impacts the quality and safety of aquatic products. Traditional aquaculture often involves the excessive use of antibiotics, chemical disinfectants and other drugs, which can lead to drug residues in aquatic products that pose a threat to consumer health. As a natural antibody, IgY has several key advantages: it is non-toxic, leaves no drug residues and does not pollute the environment (El-Kafrawy et al., 2023; Indhuprakash et al., 2024). When aquatic animals contract diseases, metabolic disorders result in the loss of nutrients such as proteins and amino acids. IgY prevents and controls diseases, ensuring normal growth and metabolism in aquatic animals. Additionally, odourless and nonirritating, IgY causes no change to the flavour profile when included in feed formulations. Sensory evaluations show that there are no significant differences in odor, taste, or meat firmness between aquatic products treated with IgY and control groups (Ehsani et al., 2019). In fact, treated animals often demonstrate better health and produce meat that is more tender. IgY also effectively inhibits bacterial growth during preservation and refrigeration, extending shelf life (Zhang et al., 2015). A novel strategy for preserving fish was suggested, using the specific antimicrobial activity of IgY against two specific spoilage organisms (*Shewanella putrefaciens* and *P. fluorescens*) in refrigerated fish (Xu et al., 2012). In summary, IgY has a dominant positive influence on the quality and safety of aquatic products in aquaculture. Through standardized production processes and long-term safety studies, potential risks can be mitigated effectively, establishing IgY as a crucial green technology for ensuring the safety and enhancing the quality of aquatic products.

## 6 Conclusion

In conclusion, oral administration of IgY is the most widely adopted and convenient method in aquaculture applications, and is particularly well-suited to large-scale farming of adult fish. It directly influences mucosal immunity within the intestinal tract. Immersion treatment primarily targets fry and juvenile fish or infections affecting the body surface and gills, it requires no physical contact with the fish. This method acts on both the skin and gill mucosa, making it ideal for high-density hatchery operations. Injection is the method that yields the swiftest efficacy and highest bioavailability, as IgY enters body fluids directly. This makes it optimal for emergency disease treatment or targeted immunization.

IgY has many structural and technical advantages over IgG and has shown significant promise in preventing and controlling diseases in aquaculture. IgY antibodies are currently considered to be the best candidate for a passive immunization vaccine in aquatic animals. However, several key technical issues still need to be resolved before it can be used on a large scale in aquaculture. Future research should focus on the following areas: 1. Optimizing IgY coating technology to enhance stability and antigen-binding affinity, enabling more persistent and effective function in complex aquaculture environments. 2. Elucidating the immunological and molecular mechanisms by which IgY activates downstream signaling pathways by binding to macrophage surface receptors. 3. Investigating whether IgY improves the balance of the gut microbiome in aquatic animals by promoting the growth of beneficial bacteria while inhibiting the proliferation of harmful bacteria. Long term follow-up studies are needed to clarify the effects of IgY on gut microbiota and aquatic ecosystems across different farming cycles and dosage levels. This will establish scientific standards for the dosage and frequency of application, preventing the risk of microbial imbalance resulting from excessive use. Such research will provide the theoretical basis and technical assurance needed to scale up the use of IgY in the green control of diseases within aquaculture, thereby advancing the sustainable development of the industry.
