# Correction: Application of IgY antibodies in passive immunization of aquaculture animals

**DOI:** 10.3389/fphys.2025.1761523

**Published:** 2025-12-17

**Authors:** Hui-Long Qiu, Xiao-Min Jin, Xiao-Mei Zhang, Feng-Qin Wang, Jia-Qiang Huang, Lian-Shun Wang

**Affiliations:** 1 College of Marine Resources and Environment, Hebei Normal University of Science and Technology, Hebei Key Laboratory of Ocean Dynamics Resources and Environments, Qinhuangdao, China; 2 Zaozhuang Animal Husbandry and Fishery Development Center, Zaozhuang, China; 3 Key Laboratory of Precision Nutrition and Food Quality, Ministry of Education, Department of Nutrition and Health, China Agricultural University, Beijing, China; 4 School of Fisheries and Life Science, Dalian Ocean University, Dalian, China

**Keywords:** IgY antibodies, passive immunization, aquaculture animals, IgY, aquaculture

**Affiliation** School of Fisheries and Life Science, Dalian Ocean University, Dalian, China was erroneously given for authors **Hui-Long Qiu** and **Feng-Qin Wang**. The correct affiliation 1 is College of Marine Resources and Environment, Hebei Normal University of Science and Technology, Hebei Key Laboratory of Ocean Dynamics Resources and Environments, Qinhuangdao, China.

The original article has been updated.

